# Supplementary material for: A conceptual model on caregivers’ hesitancy of topical fluoride for their children
Source: PLoS One. 2023 Mar 22;18(3):e0282834. doi: 10.1371/journal.pone.0282834 (PMC10032489; doi:10.1371/journal.pone.0282834)
Supplement: S1 Table — (PDF) [file pone.0282834.s001.pdf]

S1 Table. Semi-structured interview guide used to collect data from caregivers reporting topical fluoride hesitancy for their child or children.

1.On a scale of 1 to 10, with "1" being not opposed at all and "10" being totally opposed, how opposed are you to topical fluoride for your child or any of your children?
2.Please tell me which of the following responses best describes you:<br>1. I absolutely want no topical fluoride for my child.<br>2. Most of the time I say no to topical fluoride for my child.<br>3. Sometimes I say no to topical fluoride for my child.<br>4. I say yes to topical fluoride for my child, but I have thought about saying no.
3.Do you make decisions about topical fluoride differently for each of your children?[a]
4.I would like for you to think about a recent time when you said no or thought about saying no to topical fluoride for your child. Think about when it was and where you were. Please tell me about that specific experience.[b]
5 How likely is your child to get a cavity?<br>1. Extremely likely<br>2. Likely<br>3. Unlikely<br>4. Extremely unlikely
6.You said your child is [likelihood] to get a cavity. How, if at all, does this play into your decision about topical fluoride?[b]
7.Tell me what you think topical fluoride does.[b]
8.On a scale of 1 to 10, with "1" being not at all confident and "10" being totally confident, how confident are you that topical fluoride can prevent your child from getting a cavity?
9.What are other ways your child can get fluoride?[b]
10.Tell me about what you think fluoride in drinking water does.[b]
11.What is your response to your child getting fluoride in their drinking water?<br>1. Strongly oppose<br>2. Somewhat oppose<br>3. Don't care either way<br>4. Somewhat favor<br>5. Strongly favor
12.Tell me about what you think fluoride toothpaste does.[b]
13.What do you think about your child using fluoride toothpaste?<br>1. Strongly oppose<br>2. Somewhat oppose<br>3. Don't care either way<br>4. Somewhat favor<br>5. Strongly favor
14.On a scale of 1-10, with "1" being low trust and "10" being high trust, how much do you trust dentists to help you make decisions about topical fluoride?
15.What affects your trust level in dentists?[b]
16.On a scale of 1-10, with "1" being low trust and "10" being high trust, how much do you trust dentists to help you make decisions about topical fluoride?

17.What affects your trust level in doctors?[b]
18.During your child's dentist or doctor visits, how much do you discuss the treatments that your child may receive during that visit?[b]
19.Where do you get your information about topical fluoride?[b]
20.How often do you share information about topical fluoride with other parents?[b]

[a] For caregivers with more than one child.

[b] Open-ended question.
