# Supplementary material for: A conceptual model on caregivers’ hesitancy of topical fluoride for their children
Source: PLoS One. 2023 Mar 22;18(3):e0282834. doi: 10.1371/journal.pone.0282834 (PMC10032489; doi:10.1371/journal.pone.0282834)
Supplement: S2 Table — (PDF) [file pone.0282834.s002.pdf]

S2 Table. Domains, categories, and corresponding definitions for a conceptual model of topical fluoride hesitancy.

Domain	Domain Definition	Category	Category Definition
**1. Thinking topical fluoride is unnecessary**	Categories in this domain relate to caregivers' thoughts on why fluoride is unnecessary to keep their child's teeth healthy.		
		1a. Thinking my child's teeth are fine without topical fluoride	Caregivers believed topical fluoride wasn't necessary if their child didn't have permanent teeth or dental issues.
		1b. Thinking topical fluoride is not effective	Caregivers thought that topical fluoride was not going to improve the oral health of their child. Based on research and first-hand experiences with topical fluoride, they believe that topical fluoride was not effective in preventing cavities.
		1c. Keeping your teeth clean is enough	Caregivers spoke about practicing good dental hygiene instead of using topical fluoride to keep their child's teeth healthy. This included making sure their child brushed, flossed, and had professional dental cleanings.
		1d. Having a healthy diet is more important than topical fluoride	Caregivers attributed good oral health to eating healthy foods rather than to topical fluoride. They spoke about eating organic foods and limiting sugar in their child's diet to prevent cavities.
		1e. Getting fluoride from other sources is enough	Caregivers believed topical fluoride was unnecessary to keep their child's teeth healthy because they were getting enough fluoride from other sources, such as toothpaste and water.

**2. Wanting to keep chemicals like fluoride out of my child’s body**	Categories in this domain reflect caregivers' desire to keep their child's body free from unnatural chemicals, such as fluoride. They worried about their child getting too much fluoride and ingesting it.		
		2a. Being careful about what goes into my child's body	Caregivers spoke about not wanting to expose their child to synthetic chemicals, including fluoride. Natural alternatives were perceived as safer and therefore preferable.
		2b. Worrying about my child ingesting topical fluoride	Caregivers worried about their child swallowing fluoride. They were concerned about topical fluoride absorbing and accumulating in their child’s body over time.
		2c. Not wanting my child to have too much fluoride	Caregivers spoke about not wanting their child to be exposed to excessive amounts of fluoride.
**3. Thinking fluoride is harmful**	Categories in this domain reflect caregivers’ concerns about the negative health consequences, including physical, cognitive emotional consequences, for their child.		

		3a. Believing fluoride is dangerous for my child's health	Caregivers spoke about how fluoride may be harmful to general health, including negative effects on long-term health. Caregivers were concerned that it was a poison or toxin, especially in large quantities.
		3b. Believing fluoride will damage the body	Caregivers were concerned about the specific negative effects of fluoride on their child's body, including cancer and fluorosis. Concerns also included damage to the digestive, endocrine, skeletal, renal, and nervous.
		3c. Fearing fluoride will affect my child's developing mind	Caregivers feared fluoride could negatively affect their child's psychological development, particularly their cognitive and emotional development.
		3d. Worrying topical fluoride will upset my child	Caregivers spoke about their child's negative physical, emotional, and/or behavioral responses to getting topical fluoride. They spoke about saying no to topical fluoride to avoid these negative experiences for their child.
**4. Thinking there is too much uncertainty about fluoride**	Categories in this domain reflect how caregivers' uncertainty about fluoride, including a lack of knowledge about fluoride, thinking they are taking a risky gamble with their child's present and future health.		
		4a. Feeling like I don't know enough about fluoride	Caregivers spoke about feeling uninformed or confused about the purpose, benefits, and risks of fluoride. They felt like they were not provided enough information to make an informed decision about it.
		4b. Hearing negative or conflicting things about fluoride	Caregivers spoke about reading or hearing negative or conflicting information about fluoride, particularly from research studies and doctors. They spoke about how this

			made them feel uncertain about the safety and effectiveness of fluoride. Caregivers also considered their family members opposition to fluoride.
		4c. Worrying fluoride has unknown effects	Caregivers spoke about being fearful about what is still unknown about fluoride and how that might impact their child in the future. They spoke about there not being enough proof that fluoride will not do harm.
		4d. Erring on the side of caution	Caregivers believed that not using topical fluoride for their child was the safest option. While some caregivers acknowledged the benefits of topical fluoride, they did not believe that these benefits outweighed the potential risks.
**5. Feeling pressured to get topical fluoride**	Categories in this domain reflect how caregivers feel pressured to use topical fluoride, which leads them to distrust health professionals and, as a result, become more hesitant about topical fluoride. Caregivers felt that dental, pharmaceutical, and government entities coerced them to get fluoride, had an incentive to push it, and were not open about the potential risks.		

		5a. Not getting the whole truth about topical fluoride	Caregivers felt that dentists' beliefs and knowledge about topical fluoride were biased and therefore not trustworthy. Caregivers worried that they did not have enough information to make an informed decision about topical fluoride because dentists only told them the benefits of topical fluoride and not the risks. Some caregivers felt that dentists were not questioning the use of fluoride in dentistry or staying current with new information.
		5b. Getting topical fluoride pushed on me	Caregivers spoke about feeling like topical fluoride is not presented as a choice. They spoke about dentists applying topical fluoride to their child's teeth without asking and coercing them to say yes to topical fluoride. This led caregivers to distrust dentists and become more opposed to topical fluoride.
		5c. Feeling like topical fluoride comes with an agenda	Caregivers spoke about feeling like dentists and institutions, such as the government and pharmaceutical companies, had nefarious motives to promote topical and other forms of fluoride. Many caregivers described financial incentives, while some spoke of racially motivated exploitation.
**Feeling fluoride should be a choice**	Categories in this domain reflect how caregiver's value their right to make health care decisions for their child. They viewed fluoride as choice and highlighted community water fluoridation as infringing on their right to make that choice. Although caregivers		

	ultimately made the decision about topical fluoride for their child, they shared that their child’s preferences influenced that decision.		
		6a. Having the right to decide what is best for my child	As experts of their child, caregivers felt that they should have a right to decide whether to expose their child to fluoride. They spoke about how community water fluoridation infringed upon their right to decide what is best for their individual child.
		6b. Considering my child’s opinion about topical fluoride	Caregivers considered their child’s opinion about getting topical fluoride. This guided their decision to accept it or decline it at dental visits. They spoke about their older child being able to make decisions on their own and choosing not to get topical fluoride because their child did not like the taste or texture of it.
