# Supplementary material for: A conceptual model on caregivers’ hesitancy of topical fluoride for their children
Source: PLoS One. 2023 Mar 22;18(3):e0282834. doi: 10.1371/journal.pone.0282834 (PMC10032489; doi:10.1371/journal.pone.0282834)
Supplement: Attachment — Submitted filename: Response to Reviewers.docx [file pone.0282834.s004.docx]

**Responses to the editor’s and reviewers’ comments are bolded.**

Reviewers' comments:

Reviewer's Responses to Questions

**Comments to the Author**

1. Is the manuscript technically sound, and do the data support the conclusions?

The manuscript must describe a technically sound piece of scientific research with data that supports the conclusions. Experiments must have been conducted rigorously, with appropriate controls, replication, and sample sizes. The conclusions must be drawn appropriately based on the data presented.

Reviewer #1: Yes

Reviewer #2: Yes

2. Has the statistical analysis been performed appropriately and rigorously?

Reviewer #1: N/A

Reviewer #2: N/A

3. Have the authors made all data underlying the findings in their manuscript fully available?

The [PLOS Data policy](https://urldefense.com/v3/__http:/www.plosone.org/static/policies.action*sharing__;Iw!!K-Hz7m0Vt54!gD2VuSfRnP8GpoMD-43uKG4pq5PhSxl-DVhiLMVFkI3ck8PIhL9MO4lvp3CZAsJIhZe6I9WANPZj$) requires authors to make all data underlying the findings described in their manuscript fully available without restriction, with rare exception (please refer to the Data Availability Statement in the manuscript PDF file). The data should be provided as part of the manuscript or its supporting information, or deposited to a public repository. For example, in addition to summary statistics, the data points behind means, medians and variance measures should be available. If there are restrictions on publicly sharing data—e.g. participant privacy or use of data from a third party—those must be specified.

Reviewer #1: Yes

Reviewer #2: No

**We are not able to share the qualitative interview scripts because the data contain potentially identifying or sensitive patient information. We have provided institutional contact information to which data requests may be sent.**

4. Is the manuscript presented in an intelligible fashion and written in standard English?

PLOS ONE does not copyedit accepted manuscripts, so the language in submitted articles must be clear, correct, and unambiguous. Any typographical or grammatical errors should be corrected at revision, so please note any specific errors here.

Reviewer #1: Yes

Reviewer #2: Yes

5. Review Comments to the Author

Please use the space provided to explain your answers to the questions above. You may also include additional comments for the author, including concerns about dual publication, research ethics, or publication ethics. (Please upload your review as an attachment if it exceeds 20,000 characters)

Reviewer #1: Could not access Appendix A as referred to in the text. Received no response from Plos One when requested this. Definitions are required of methods used and references citations need to corrected. See other comments on attached feedback.

**We apologize for this oversight. Both Appendix A and B have been provided as Supplementary Table 1 and Supplementary Table 2. Revisions have been made to address concerns related to the Methods section and reference citations. We have also made revisions based on comments on the PDF. Thank you very much for the careful review.**

Reviewer #2: This is a well-written article on an important topic in clinical dentistry. The methods are sound and the data are presented well. It was an enjoyable read.

The authors state that some carers have conspiracy theory type views, whereas others’ hesitancy could really be consistent with clinical guidelines anyway. What will help the general reader is a clear statement of the current guidelines for topical fluoride application, presumably from Reference 46 or the American Dental Association or equivalent bodies. Furthermore, what is the current practice with fluoride application at the study site? Is topical fluoride recommended for all children? Do clinicians take steps that are necessary to prevent fluorosis?

**Thank you for this clarifying point. The relevant part of the Discussion section has been expanded to make it clear that the American Academy of Pediatric Dentistry recommends topical fluoride for children at risk for caries (and not all children). Language was included that almost all clinics offer topical fluoride to all children. Topical fluoride is not recommended for all children. Fluorosis is related to chronic ingestion of fluoride, and therefore the topical fluoride provided during medical and dental visits is not considered a risk factor.**

The text in the two paragraphs of the Discussion on reshaping dental practitioner behavior (p22-23) cover important contents and should appear earlier on. A long explanation (discussion) carries the risk of such important messages being lost in the details. Some of the text on future studies could appear much later. A more succinct explanation could also help to keep readers interested.

**We agree the discussion points about reshaping providers’ behaviors is important. However, we wanted to place these points after discussing the main findings related to the conceptual model, which was the primary aim of the study. Therefore, placement of these paragraphs was not altered. We agree parts of the discussion section could be cut to reduce wordiness and keep readers interested. These changes are reflected in the revised paper.**

An important area to explore would be mapping carer’s attitude with clinical data on caries risk and provider’s recommendation for topical fluoride application. I realise this is probably out of the scope of the current project, in which case the limitation should be acknowledged in the Discussion section.

**Unfortunately, we do not have clinical data on the children. But this is an excellent point and is the topic of future investigations. This is included as a study limitation.**

I have made some more relatively minor suggestions below.
P3, last paragraph: The sentence ‘Patients have also increasingly scrutinized providers for promoting treatments that generate income [33]’ could be better expressed using the term ‘overtreatment’. It is not only patients but also the general public, academics and regulatory bodies that are scrutinizing overtreatment, so the sentence could be better written as, “There is also an increasing scrutiny over overtreatment ….”.

**We agree. This has been fixed as suggested.**

P5: [9, 35-36]

**This has been fixed.**

P17, Domain 5: … to question its necessity…

**Done.**

P19: Non-US readers may not understand what AIAN means, so please use its full form with abbreviation in brackets the first time it appears in text. Alternatively, if it does not appear elsewhere in the text, please just use the full form.

**Great point. This has been revised.**

P21: Please simplify this sentence as it is a bit too long, “Such an approach might be interpreted by some caregivers as being insensitive, but mis-specified
education could also come across as dismissive or make hesitant caregivers feel like they are
being pressured to accept fluoride, leading to reactance.”

**This has been broken into 2 sentences.**

References: Please ensure formatting consistency, e.g., sentence case or first letter capitalization for title, and inclusion or exclusion of publication date.

**Done.**

Reference 41 – please insert web-link.

**Done.**

6. PLOS authors have the option to publish the peer review history of their article ([what does this mean?](https://urldefense.com/v3/__https:/journals.plos.org/plosone/s/editorial-and-peer-review-process*loc-peer-review-history__;Iw!!K-Hz7m0Vt54!gD2VuSfRnP8GpoMD-43uKG4pq5PhSxl-DVhiLMVFkI3ck8PIhL9MO4lvp3CZAsJIhZe6I4yDb75y$)). If published, this will include your full peer review and any attached files.


If you choose “no”, your identity will remain anonymous but your review may still be made public.


**Do you want your identity to be public for this peer review?** For information about this choice, including consent withdrawal, please see our [Privacy Policy](https://urldefense.com/v3/__https:/www.plos.org/privacy-policy__;!!K-Hz7m0Vt54!gD2VuSfRnP8GpoMD-43uKG4pq5PhSxl-DVhiLMVFkI3ck8PIhL9MO4lvp3CZAsJIhZe6I2GgYpVE$).

Reviewer #1: No

Reviewer #2: No
